# Supplementary material for: Mechanical dissipation from charge and spin transitions in oxygen-deficient SrTiO3 surfaces
Source: Nat Commun. 2018 Jul 27;9:2946. doi: 10.1038/s41467-018-05392-1 (PMC6063934; doi:10.1038/s41467-018-05392-1)
Supplement: Supplementary file 1 — Supplementary Information [file 41467_2018_5392_MOESM1_ESM.pdf]

Supplementary information for: Mechanical dissipation from charge and spin transitions in oxygen deficient $SrTiO_3$

by Kisiel *et al.*

SUPPLEMENTARY METHODS

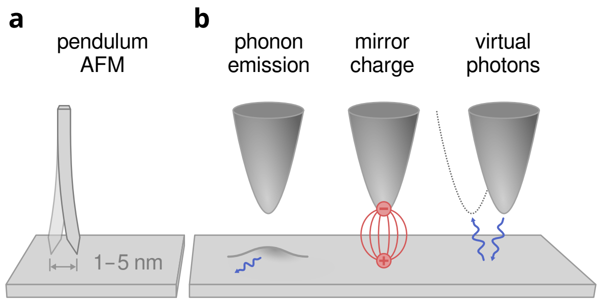

Supplementary Figure 1: Pendulum AFM (**a**) A sketch of the AFM cantilever suspended in pendulum geometry. (**b**) Origins of energy dissipation at the nanoscale.

Pendulum UHV-AFM is a home-built design dedicated to perform measurements of extremely small forces. In the pendulum geometry the cantilever is hoovering perpendicularly to the sample surface, shearing the nanometer vacuum gap between tip and the sample. In this arrangement very soft cantilevers with spring constants of $k \approx 10^{-5} - 10^{-3}$ Nm^{-1} can be used as force sensors, avoiding snapping into the contact with the sample. The force sensitivity is equal to: $F_{\text{min}} = \sqrt{\frac{2k_{\text{B}}Tk}{\pi f Q}}$, which for ultra-sensitive cantilevers is on the level of $\frac{aN}{\sqrt{Hz}}$. Pendulum AFM operates at cryogenic temperatures T=5K and typical quality factors are in the order of $Q \approx 10^5 - 10^6$. High Q, together with extremely small k imply that minimal detectable energy dissipation is in the order of: $P = \frac{\pi k A^2}{eQ} \approx \frac{\mu eV}{cycle}$, few orders of magnitude smaller as compared to the standard AFM configuration.

Three main dissipation mechanisms might take place between the oscillating tip and the sample: phononic friction occurs when the surface deformation is dragged by the moving tip and the energy is lost to the creation of longitudinal phonons. Joule dissipation originates due to creation of local currents induced when charged tip hoovers on top of resistive media. Van der Waals friction arises from the surface dielectric fluctuations. The dissipation occurs when moving tip experiences different electromagnetic environment every oscillating cycle.

SUPPLEMENTRY FIGURES

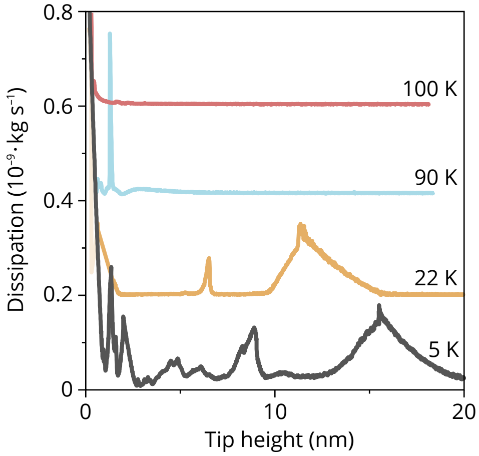

Supplementary Figure 2: Temperature dependence of AFM dissipation spectra on reduced $SrTiO_3$. Tip-sample-distance dependence of energy dissipation for different temperatures ranging from 5K to 100K taken at the same bias voltage $V = 1$ V. Due to the thermal drift and linear elongation of the scanner tube different temperatures also correspond to different spatial tip locations on the surface which explains the different position of the peaks. Please note that for temperature $T = 100$K the dissipation peak fully vanishes.The distance $z = 0$ corresponds to the point where the tip enters the contact regime, meaning that the cantilever driving signal is saturated.

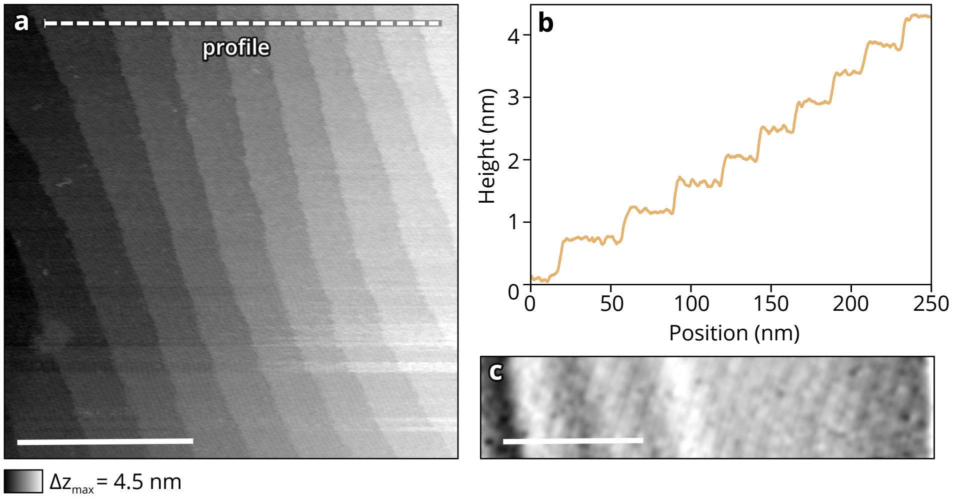

Supplementary Figure 3: Large scale surface characterisation with STM. Constant current STM images of $SrTiO_3$ surface long-term annealed at $T = 950°C$ taken with a metallic tip in pendulum geometry. The tunneling parameters were $U_{tip} = -1.5$ V, $I = 100$ pA and $U_{tip} = -0.55$ V, $I = 30$ pA for (**a**) and (**c**), respectively. The lenght of the scale bar is equal to 100nm and 5nm on (**a**) and (**c**), respectively. Height profile (**b**) shows that the surface is composed of atomically flat terraces of average width about 25 nm. Dark features visible on (**c**) are presumed to be oxygen vacancies. Note that already after annealing to 950°C samples are morphologically close to that shown in Fig. 1 (**b,c**).

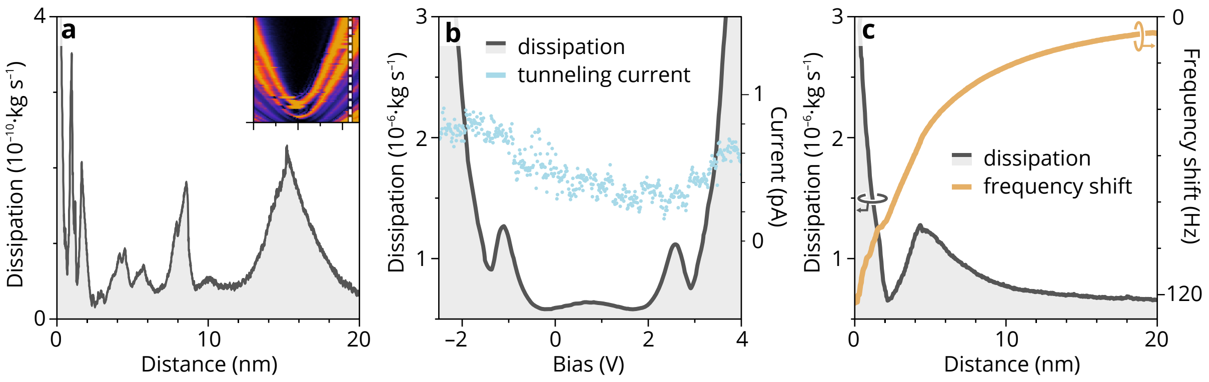

Supplementary Figure 4: Distance and voltage dependent AFM dissipation. Dissipation (friction coefficient Γ) was measured with an ultra-sensitive soft Atec-Cont cantilever probe oscillating at large amplitudes (**a**) and a with very stiff tuning fork sensor (**b**), (**c**). Bias dependence of dissipation with the stiff tip is shown in (**b**), together with simultaneously monitored tunneling current. Please note that tunneling current is below minimum detectable value of $I = 500$ fA. On (**c**) distance dependent dissipation is shown together with tuning fork frequency shift, which increases in magnitude as the tip approaches the sample surface. The sharp dissipation increase is accompanied by a discontinuity in the force spectra, similar to that observed for semiconductor quantum dots [1].

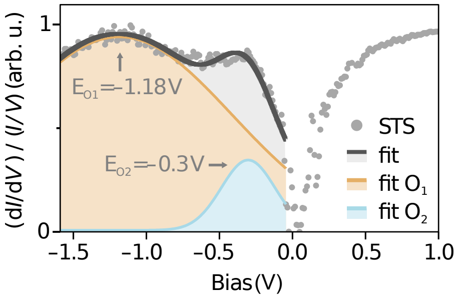

Supplementary Figure 5: Shallow vacancy level characterisation with STS. Normalised differential conductance $(dI/dV)/(I/V)$ is approximately proportional to the electronic density of states and a clearer picture of the electronic vacancy gap-states' positions. Here the normalized conductance was measured in constant-height mode using lock-in technique (dots). The modulation frequency and voltage were equal to 653 Hz and 15 mV, respectively. The two electronic states located at $E_{01} = 1.18$ V and $E_{02} = 0.3$ V below E_{F} are visible and their position was established by Gaussian fit to the measured data. The deeper of the states is presumed to be a signature of the oxygen-defficient and reconstructed surface of STO [2] while the shallow state at 0.3 eV we attribute to the single vacancy gap state coinciding well with theoretical predictions [3].

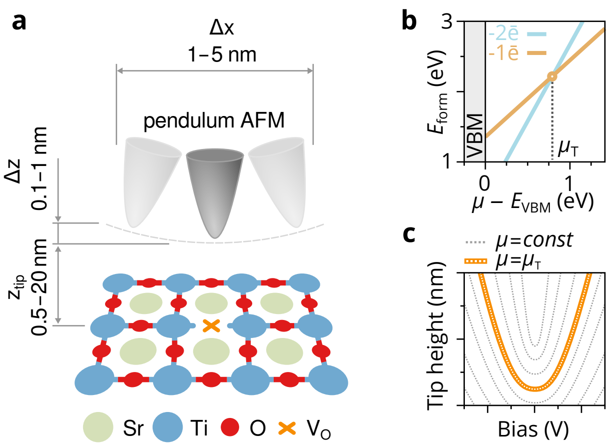

Supplementary Figure 6: Pendulum AFM as a characterization tool for oxygen vacancies on $SrTiO_3$. (**a**) A sketch of the setup: pendulum AFM tip oscillating over an oxygen vacancy on the TiO_2-terminated surface of strontium titanate. The vacancy is characterised by two dangling Ti-bonds left behind by the departing oxygen atom which then trap the two electrons also left behind by the oxygen. The system acts like a quantum dot with the two additional electrons giving each Ti a local magnetic moment of 1 μ_B. The pendulum AFM tip suspended above the surface at a range of heights starting from 0.5 nm and swinging with a lateral amplitude of $1-5$ nm and vertical amplitude of less than 1 nm can overshadow one or several vacancies (depending on the lateral amplitude). (**b**) Formation energy diagram for different charge states of a single oxygen vacancy at the TiO-terminated $SrTiO_3(001)$ surface as a function of the chemical potential μ imposed by the AFM tip (respective to the valence band maximum). (**c**) Sketch explaining the observed dissipation parabolas. In the AFM-tip height-bias space lines of constant exerted chemical potential form slightly distorted parabolas. If the chemical potential exerted by the AFM tip coincides with the transition potential for an oxygen vacancy, periodic V_O charge and spin state transitions present a dissipation channel for the AFM pendulum oscillation energy, resulting in a dissipation peak as seen on the dissipation map presented in Fig. 4(**a**).

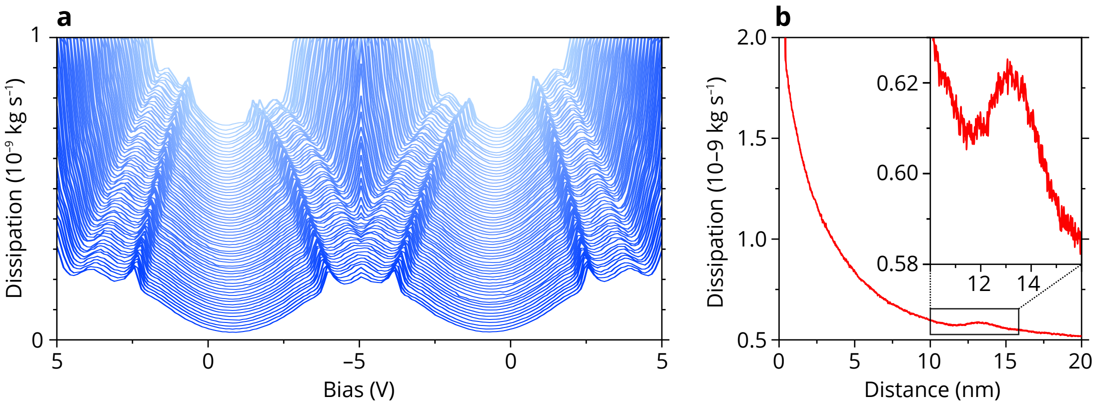

Supplementary Figure 7: Dissipation map (a) and distance dependent dissipation single spectra (b). Data shown in (**a**) correspond to dissipation map shown in Fig. 4(**a**) of the main manuscript. On (**a**) bias voltage was swept forward and backward. On (**b**) tip sample voltage was kept constant and equal to $U = 3$ V.

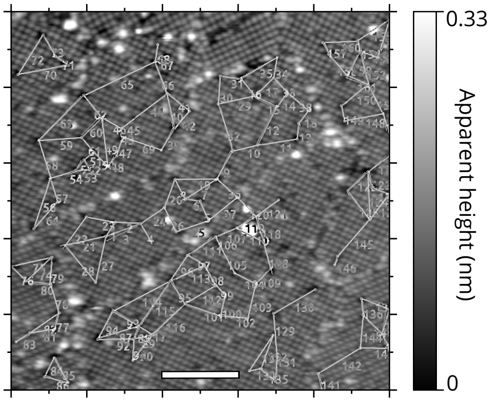

Supplementary Figure 8: Spatial distribution of oxygen vacancies on the surface of reduced $SrTiO_3$. Constant current STM image of the $SrTiO_3$ surface long-term-annealed at $T = 1050°C$. The tunneling parameters were $U_{tip} = -1$ V, $I = 10$ pA. Dark features visible in strongly reduced samples after high temperature annealing are related to oxygen vacancies and the average distance between nearest oxygen vacancies dark defects was calculated to be equal to 3.7 ± 1.6 nm. The length of the scale bar is equal to 10 nm.

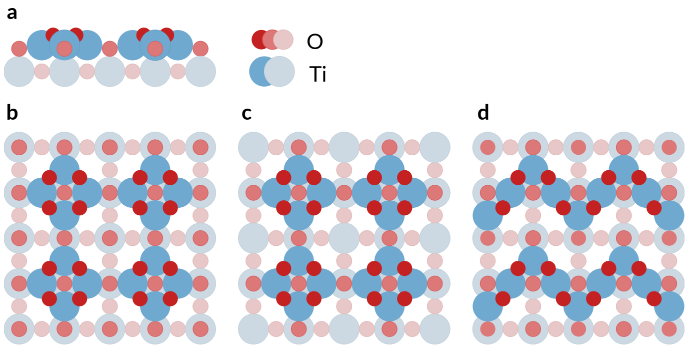

Supplementary Figure 9: Theoretically studied 2×2 reconstructed $SrTiO_3$ surface configurations. We have calculated the electronic structure of single oxygen vacancies at a reconstructed $SrTiO_3$ (001) surface, choosing as a test subject the example of three known 2×2 reconstruction patterns. [4, 5] We find that including the reconstruction into consideration does not qualitatively alter the the behavior of the V_O– with changing imposed chemical potential the vacancy goes through a series of charge transitions which shall result in a dissipation channel for an AFM tip imposing the chemical potential change.

[1] Cockins, L. *et al.* Energy levels of few-electron quantum dots imaged and characterized by atomic force microscopy. *Proceedings of the National Academy of Sciences* **107**, 9496–9501 (2010).

[2] Tanaka, H., Matsumoto, T., Kawai, T. & Kawai, S. Surface Structure and Electronic Property of Reduced $SrTiO_3$ (100) Surface Observed by Scanning Tunneling Microscopy/Spectroscopy. *Japanese Journal of Applied Physics* **32**, 1405–1409 (1993).

[3] Brovko, O. O. & Tosatti, E. Controlling the magnetism of oxygen surface vacancies in $SrTiO_3$ through charging. *Physical Review Materials* **1**, 044405 (2017).

[4] Shiraki, S., Nantoh, M., Katano, S. & Kawai, M. Nanoscale structural variation observed on the vicinal $SrTiO_3$(001) surface. *Applied Physics Letters* **96**, 231901 (2010).

[5] Lin, Y. *et al.* The (2×2) reconstructions on the $SrTiO_3$ (001) surface: A combined scanning tunneling microscopy and density functional theory study. *Surface Science* **605**, L51–L55 (2011).
